# Mesenchymal cell interaction with ovarian cancer cells induces a background dependent pro-metastatic transcriptomic profile

**DOI:** 10.1186/1479-5876-12-59

**Published:** 2014-03-05

**Authors:** Raphael Lis, Cyril Touboul, Najeeb M Halabi, Abishek Sainath Madduri, Denis Querleu, Jason Mezey, Joel A Malek, Karsten Suhre, Arash Rafii

**Affiliations:** 1 Department of Genetic Medicine and Obstetrics and Gynecology, Stem cell and microenvironment laboratory, Weill Cornell Medical College in Qatar (WCMC-Q), Education City, Qatar Foundation, Qatar-Foundation PO: 24144, Doha, Qatar; 2 Department of Genetic Medicine, Weill Cornell Medical College, New York, NY, USA; 3 Institut Claudius Regaud, Toulouse F-31052, France; 4 Genomic Core, Weill Cornell Medical College in Qatar (WCMC-Q), Education city, Qatar Foundation, Doha, Qatar; 5 Department of Physiology and Biophysics, Weill Cornell Medical College, New York, NY, USA; 6 Department of Gynecologic Surgery, Hospital Arnaud de Villeneuve, CHU, Montpellier, France

**Keywords:** Ovarian cancer, Mesenchymal stem cell, Transcriptome, Genomic modification, Metastasis

## Abstract

**Background:**

The cross talk between the stroma and cancer cells plays a major role in phenotypic modulation. During peritoneal carcinomatosis ovarian cancer cells interact with mesenchymal stem cells (MSC) resulting in increased metastatic ability. Understanding the transcriptomic changes underlying the phenotypic modulation will allow identification of key genes to target. However in the context of personalized medicine we must consider inter and intra tumoral heterogeneity. In this study we used a pathway-based approach to illustrate the role of cell line background in transcriptomic modification during a cross talk with MSC.

**Methods:**

We used two ovarian cancer cell lines as a surrogate for different ovarian cancer subtypes: OVCAR3 for an epithelial and SKOV3 for a mesenchymal subtype. We co-cultured them with MSCs. Genome wide gene expression was determined after cell sorting. Ingenuity pathway analysis was used to decipher the cell specific transcriptomic changes related to different pro-metastatic traits (Adherence, migration, invasion, proliferation and chemoresistance).

**Results:**

We demonstrate that co-culture of ovarian cancer cells in direct cellular contact with MSCs induces broad transcriptomic changes related to enhance metastatic ability. Genes related to cellular adhesion, invasion, migration, proliferation and chemoresistance were enriched under these experimental conditions. Network analysis of differentially expressed genes clearly shows a cell type specific pattern.

**Conclusion:**

The contact with the mesenchymal niche increase metastatic initiation and expansion through cancer cells’ transcriptome modification dependent of the cellular subtype. Personalized medicine strategy might benefit from network analysis revealing the subtype specific nodes to target to disrupt acquired pro-metastatic profile.

## Background

Ovarian cancer is the most deadly gynecologic cancer with most patients dying from diffuse peritoneal disease [1]. Defined steps involving changes of cancer cells’ phenotypes are implicated in the development of peritoneal carcinomatosis (reviewed in [2]). Many studies in the literature illustrate the existence of a cross talk between cancer and stromal cells [3-5]. There are evidences that these interactions modulate cancer cells’ phenotype and become more invasive.

Among the different cell lines playing a role in tumor microenvironment, mesenchymal stem cells (MSC) have been widely studied. They are actively recruited at the site of metastasis and their interactions with cancer cells increase their metastatic potential. The modulation of phenotype through cross-talk has been illustrated for ovarian cancer. Indeed upon their interaction with mesenchymal cells ovarian cancer increase their migration, metastasis and resistance to chemotherapy or hyperthermia [5-10].

Most studies focus on specific factors in the acquisition of a metastatic profile such as a cytokine or a membrane bound factor but the translation of these findings to the clinical setting has been quite disappointing [5,10]. We can hypothesize that change of phenotype is most probably underlined by broad transcriptomic and epigenetic changes. Only few studies are assessing global transcriptomic changes occurring in cancer cells upon their interaction with MSC [3]. Zhang S et al. [3] reported modification of the prostate cancer cells transcriptome induced by interaction with MSCs toward a new pro-metastatic state. Understanding the pathways impacted by the interaction of cancer cells and their microenvironment and the induced global modifications is mandatory in order to be able to target microenvironment specific pathways [3].

Our knowledge of ovarian cancer biology has evolved. The broad TCGA study demonstrated the heterogeneity of serous ovarian cancer with at least 4 different subtypes [11]. We have illustrated the genetic (copy number variation) and genomic (gene expression) heterogeneity between primary and metastatic disease [12,13]. We must therefore understand the interaction between cancer and stromal taking into account tumor heterogeneity and plasticity.

We have recently shown that the interaction between cancer cells and MSC induced a pro-metastatic phenotype with an increase in cell adherence, invasion, proliferation and chemoresistance [14]. The two different ovarian cancer cell lines (OCC) used OVCAR3 and SKOV3 represent an epithelial and mesenchymal subtype respectively. Here in this follow-up study, using Ingenuity pathway analysis we demonstrate that genes related to cellular adherence, invasion, migration, proliferation and chemoresistance are modified upon OCC/MSC contact in a cell line specific manner. Our results suggest that different specific pathways may be targeted to disrupt the acquired pro-metastatic profile.

## Methods

### Cell culture

We used two ovarian cancer cell (OCC) lines - SKOV3 (HTB-77) and OVCAR3 (HTB-161) - purchased from ATCC. They were cultured in DMEM high glucose [Hyclone, Thermo Scientific], 10% FBS [Hyclone, Thermo Scientific], 1% Penicillin-Streptomycin-Amphotericyn B solution [Sigma], 1X Non Essential Amino-Acid [Hyclone, Thermo Scientific], following ATCC recommendations. Mesenchymal cells were purchased from Stem Cells (BM-MSC), Inc (Vancouver, CA, catalog number 70022) maintained and expanded in culture using MesenCult® MSC Basal Medium completed with Mesenchymal Stem Cell Stimulatory Supplements (Stem cell Inc, Vancouver, CA). Their ability to differentiate in adipocytes, osteoblasts and chondrocytes was verified as per the supplier instructions (data not shown). All cells were used at early passages (less than 10). Mycoplasma screening was conducted for all cell cultures.

### Co-cultures

We established co-cultures of eGFP-OCC (ovarian cancer cell lines cited above an transfected by a stable GFP expressing plasmid) with BM-MSC at ratio of 1:2 for 24 hours. OCC were differentiated from BM-MSC based on their eGFP and Ep-Cam. The different cell populations were sorted using Fluorescence Activated Cell Sorting (FACS). 24H was selected as the functional effects were also assessed at this timepoint.

### Fluorescent activated cell sorting

Cells were harvested and blocked in PBS-5% FBS-1% BSA-10% FcR Blocking Reagent (Myltenyi Biotec). Single-cell suspension was analyzed and sorted on SORP FACSAria2 (BD Biosciences). Data were processed with FACSDiva 6.3 (BD Biosciences). Doublets were excluded by FSC-W × FSC-H and SSC-W × SSC-H analysis, single stained channels were used for compensation, and fluorophore minus one (FMO) controls were used for gating. eGFP fluorescence was acquired with 488 nm blue laser and 510/50 nm emission, 50 000 events were acquired per sample. Charts display the median of fluorescence intensity (mfi) relative to control. During cell-sorting purity-phase mask was applied. OCC monocultures were processed and sorted as controls.

### Gene expression analysis

Upon cell sorting mRNA was isolated using Trizol reagent followed by purification using RNAeasy extraction kit from Qiagen. 200 ng of total RNA were analyzed on Affymetrix GeneChip Human Genome U133 Plus 2.0 Array. Data were analyzed using Partek software (St Louis, MO). Class comparison between different conditions (three biological replicates) was performed to identify gene expression changes with significant expression differences and two-fold increased or decreased expression. Principal component analysis (PCA) were performed using Partek with the standard settings. Statistical comparisons for microarray data were calculated using two-tailed Students t-test. Benjamini-Hochberg correction was applied to limit positive false discovery rate to 5%. Statistical comparisons for categorical data were achieved using Chi-squared test. Correlations were performed using Pearson correlation. All other statistical comparisons were calculated using two-tailed t-test.

### Ingenuity pathway analysis

We used Ingenuity Pathway Analysis software (IPA) (Ingenuity Systems, Redwood City, CA) for network analysis of genes that were differentially regulated upon co-culture. We defined global gene lists representing IPA keywords: cell adherence, migration, invasion, proliferation, chemoresistance, and apoptosis. We then constructed networks by overlaying the up and down regulated genes with these lists. In the resulting networks genes are represented as nodes, and biological relationships between two nodes as lines. All edges are supported by at least one reference from the literature, textbook, or canonical information stored in the Ingenuity Pathways knowledge database. *P-*values for enrichment of canonical pathways were generated based on the hypergeometric distribution and calculated with the right-tailed Fisher’s exact for 2 × 2 contingency tables as implemented in Ingenuity.

### Clinical gene expression comparison analysis

We compared our cell line expression data with publically available data from The *Cancer Genome* Atlas (TCGA) project (http://cancergenome.nih.gov/) (Additional file 1). This data consists of 493 ovarian cancer samples from human patients. We used normalized gene expression intensities (level 3 data) precalculated by TCGA. We calculated Pearson’s correlation coefficients and associated p-values (implemented in Matlab R2013a) between the TCGA signal intensities (493 patients) and cell line expression changes following co-culture with MSCs for all significantly varying cell line genes. In addition, we computed random correlations and p-values between randomly chosen TCGA genes and the cell line significantly varying genes to estimate the correlations randomly expected. The TGCA sample ids used are in the Additional file 1 text file and the cell line expression data is in the Additional file 2 Excel file.

## Results

### Modification of the transcriptome of OCC upon interaction with MSC

**Figure 1:**
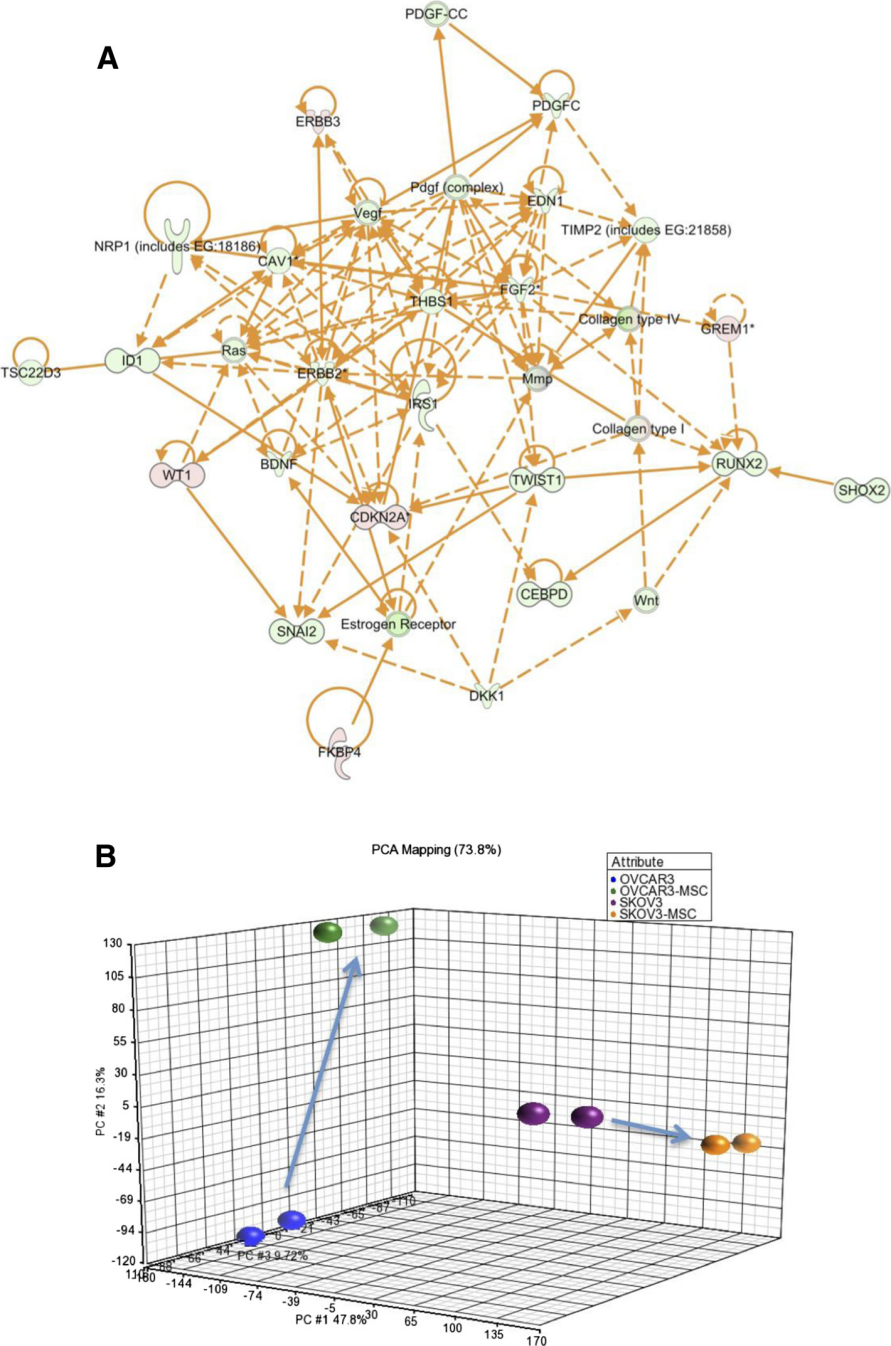
**Transcriptomic differences between OVCAR3 and SKOV3 and PCA after interaction with the mesenchymal cells. A**. Ingenuity pathway analysis network obtained when the differentially regulated genes genes between SKOV3 and OVCAR3 were overlaid on the gene list related to mesenchymal phenotype. Genes in green are over-expressed by at least 5 fold in SKOV3, genes in red are over-expressed in OVCAR3 (by at least 5 folds). **B**. PCA analysis for the ovarian cancer cells lines alone or post-contact with the Mesenchymal cells.

We compared the transcriptome of the two cell lines used in this study OVCAR3 and SKOV3. We found that 880 genes were up or downregulated over 5 fold (FDR 0.01) illustrating that the two cell lines are quite different. We looked at different set of genes and found that SKOV3 up-regulated genes related to a mesenchymal subtype (HOX (14 fold), FAP (28 fold), TWIST (9 fold), SNAIL (8 Fold)) when compared to OVCAR3, which displayed a more epithelial phenotype. PCA analysis showed that the replicates of each experimental condition “clustered” together. Gene expression pattern between all experimental conditions, were clearly distinct. Interestingly changes in the direction of gene expression upon cell contacts were distinct for both cell lines (Figure 1A and B) (Additional file 2).

IPA global analysis of differentially expressed genes for each cell line revealed significant enrichment of the category “Cancer” among the super-category “Diseases and disorders” as the most significant class. This observation indicates that upon cell contacts cancer related genes significantly change their expression pattern. Other enriched classes coherent with the experimental design included “Reproductive system disease”, “tumor morphology” and classes related to tissue development and cellular movement (Table 1). Using the genes from the “Cancer” category we built the networks presented in Additional file 3: Figure S1 and Additional file 4: Figure S2. While global analysis allows understanding of relationship between genes it is difficult to interpret when looking at particular functions. We therefore built smaller focused networks on specific metastatic traits described previously [14].

**Table 1:** Most relevant networks retrieved by IPA

**Cell line and category**	**Pathways and functions**	**p**	**Number of genes**
**OVCAR3**			
Disease and disorders			
	Cancer	3.77 10^-21^	167
	Reproductive system disease	5.58 10^-9^	105
Physiological system development and function			
	Connective tissue development and function	2.85 10^-5^	45
	Tumor morphology	1.03 10^-4^	49
**SKOV3**			
	Cancer	1.9 10^-12^	88
	Reproductive system disease	1.8 10^-10^	67
Molecular and cellular function			
	Cellular movement	1.9 10^-7^	45
	Tissue development	3.3 10^-7^	55

### Gene network associated to increased OCC migration, invasion and adherence

**Figure 2:**
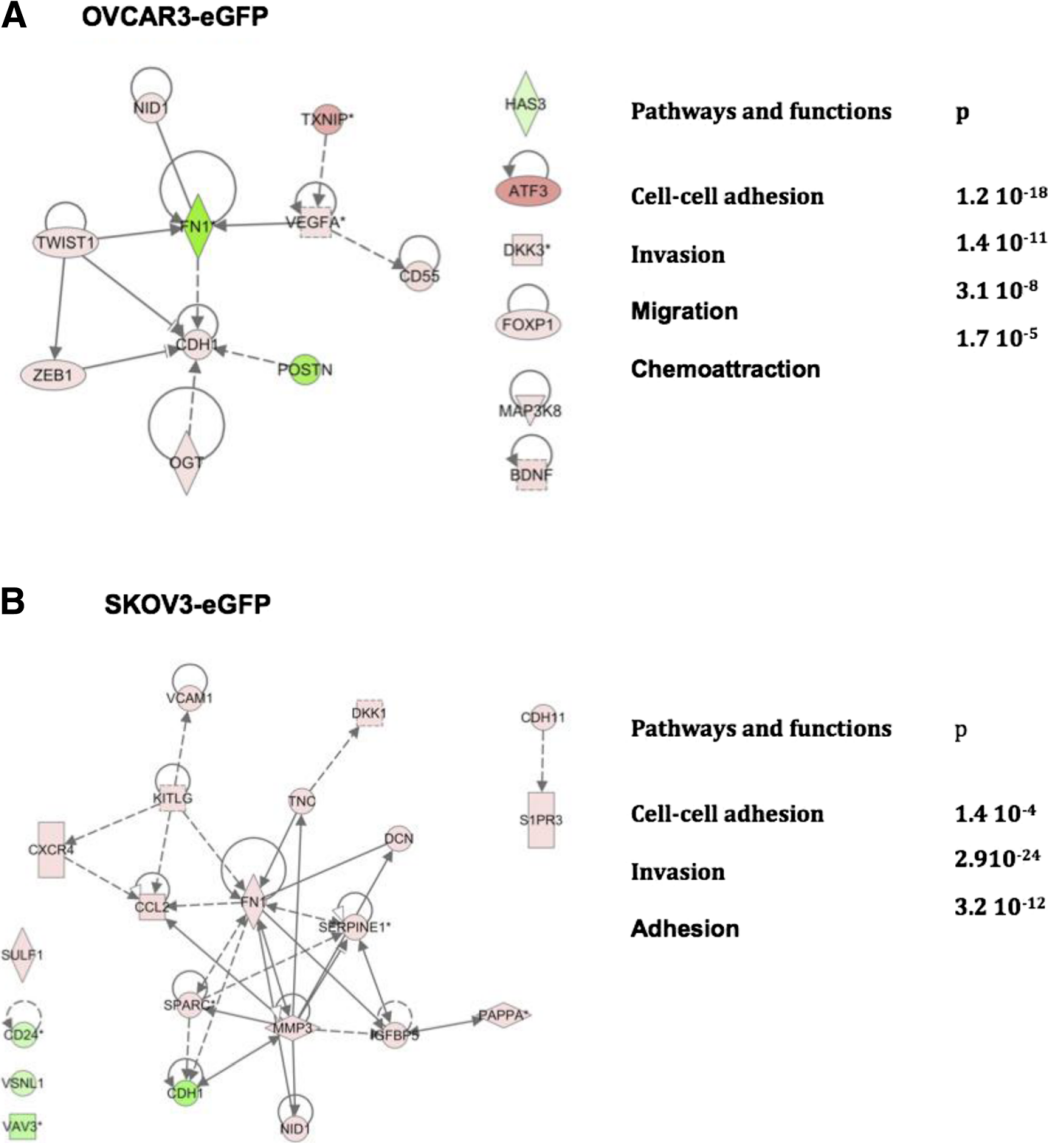
**Pathways modified in OCC upon MSC contact related to adherence, migration, and invasion. A**. Ingenuity Pathway Analysis obtained from OVCAR3-eGFP following MSC contact. **B**. Ingenuity Pathway Analysis obtained from SKOV3-eGFP following MSC contact.

We have shown previously that the interaction between MSCs and OCCs increased cell migration, invasion and adherence [14]. Genes implicated in cellular movement, adherence, migration and invasion were identified using IPA (Table 2). The genes were distinct for both cell types. In order to investigate the relationship between the involved genes we constructed cell type specific networks presented in Figure 2A and B.

**Table 2:** Lists of differentially regulated genes modified for each cell line as retrieved by IPA network analysis

**Genes**	**Fold**
**OVCAR3**	
**Adhesion, invasion and migration**	
TWIST	2.85
ZEB	2
CDH1	2.8
Hyaluronan Synthase 3	-2.7
FN1	-5.67
**Proliferation**	
CEBPB	5.3
CCND2	5.4
CDKN1C	4.2
BCL6	5.5
RASGRP1	2.17
CCNE2	-3.9
GMNN	-2
SKP2	-2.2
SPARC	-3.8
**Chemoresistance**	
GADD45A	8.7
DDIT3	9.7
NR3C1	3
ATF2	2.65
RASGRP1	2.1
**SKOV3**	
**Adhesion, invasion and migration**	
CXCR4	2.9
FN1	2
MMP3	5.8
Serpine1	3.2
PAPP-A	7.2
SPARC	4.6
CDH1	-4.4
CD24	-2.1
VAV3	-2.9
**Proliferation**	
INHBA	3.5
FN1	2
IGFBP5	4.3
SPARC	4.6
COL1A1	9.8
**Chemoresistance**	
SPARC	6.4
PDGFRA	4.5
S1PR3	2.8
KITLG	2.2
IGFBP5	4.4
SCD	-4.2
FASN	-2
DDIT4	-2.5

### MSC sustain ovarian cancer cells proliferation

**Figure 3:**
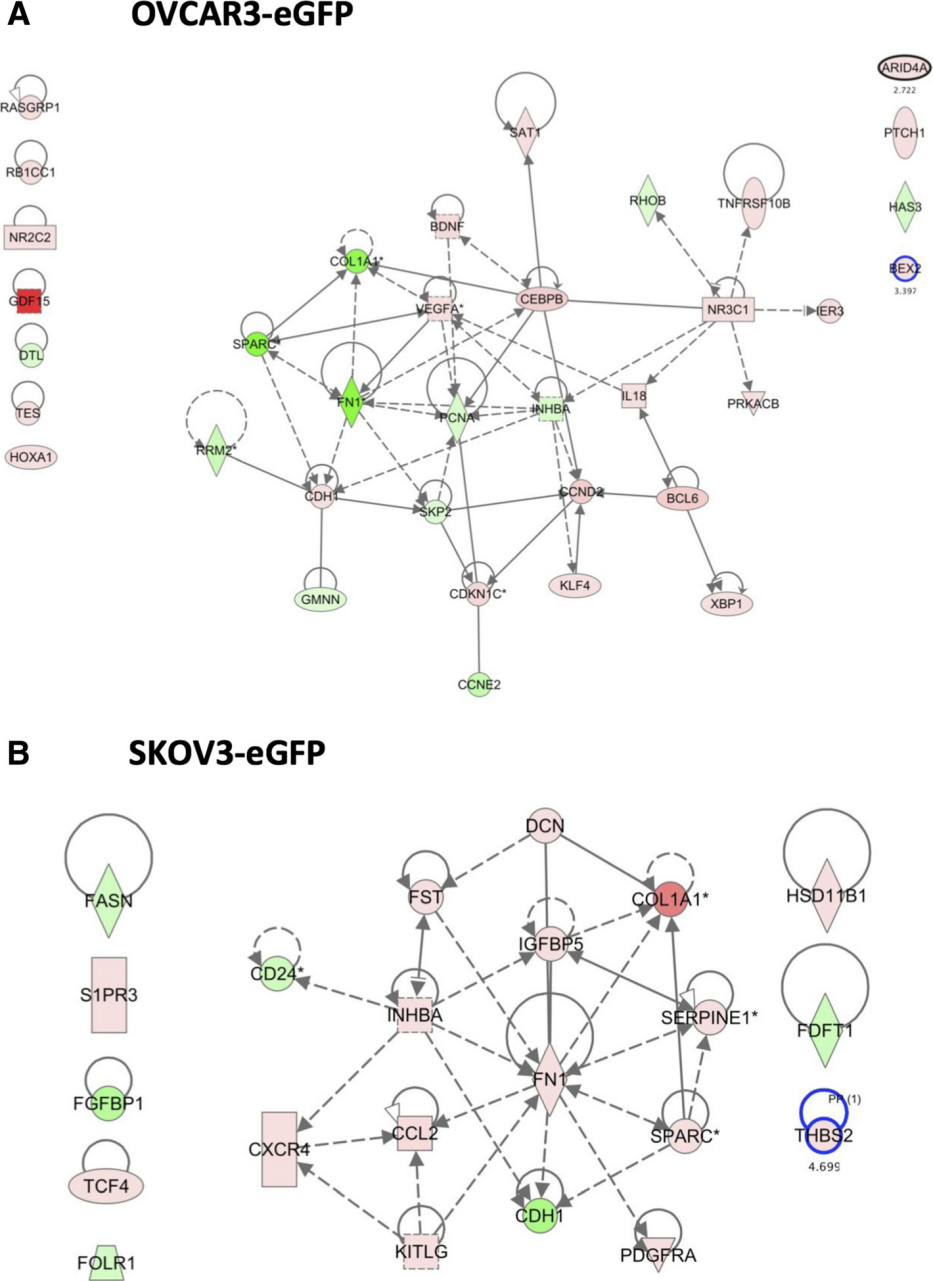
**Networks associated with increased proliferation. A**. Networks involving all “proliferation” related genes in OVCAR3 upon co-culture with MSC. **B**. Networks involving all “proliferation” related genes in SKOV3 upon co-culture with MSC.

MSC were able to sustain proliferation of OCCs for up to 15 days in a serum free context. In order to interpret these findings in the light of the expression data we constructed focused IPA networks using “proliferation” as a keyword (Figure 3A and B).

OVCAR3 and SKOV3 presented upon co-culture concomitant up-regulation of different pro-proliferation genes and down-regulation of different anti-proliferation genes. Interestingly genes such as SPARC and COL1A1 were regulated inversely in SKOV3 compared to OVCAR3, again illustrating the cell-type specificity of gene expression pattern.

### MSC induce chemoresistance of ovarian cancer cells

**Figure 4:**
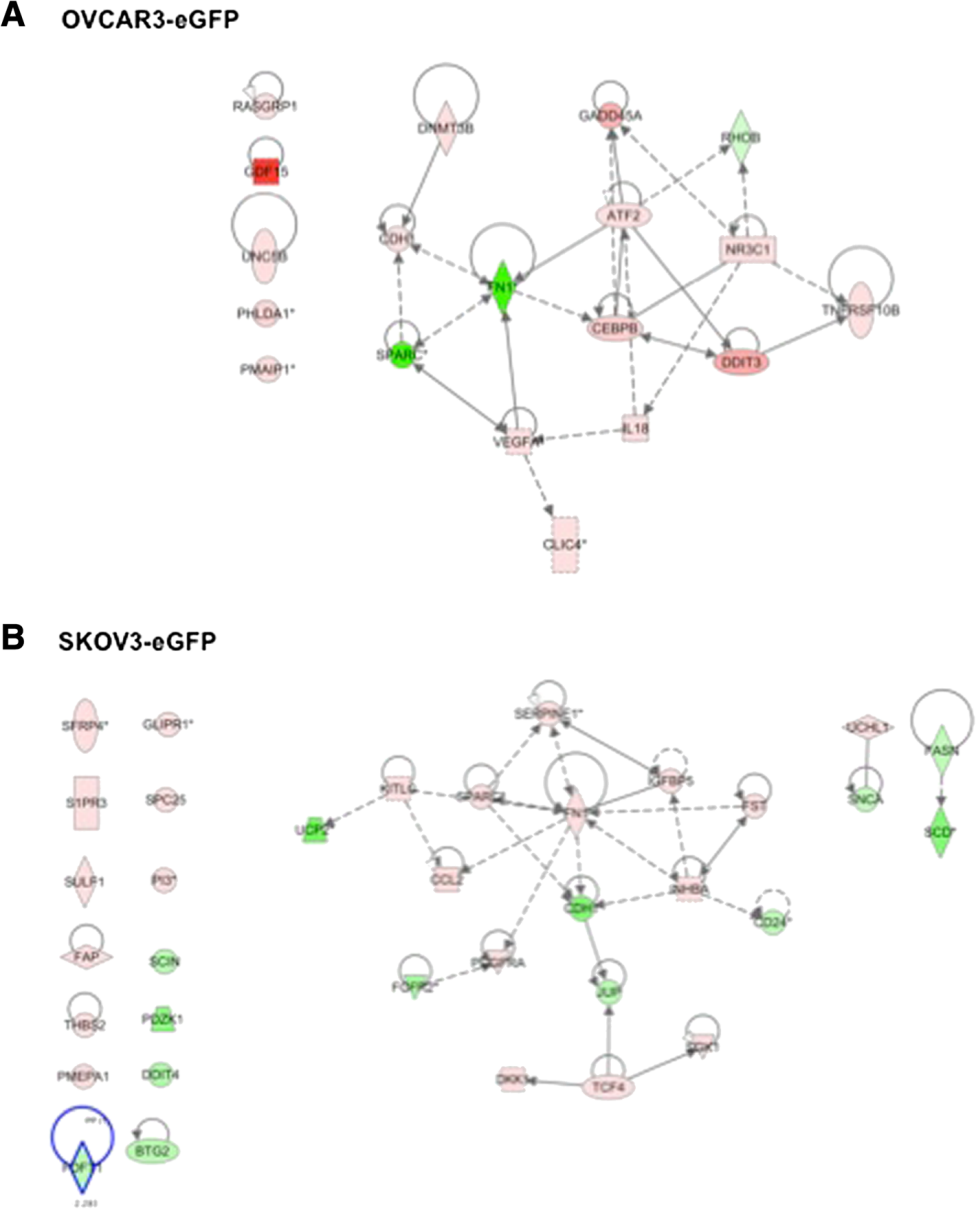
**Pathways modified in OCC upon MSC contact related to chemoresistance. A**. Ingenuity Pathway Analysis obtained from OVCAR3 following MSC contact. **B**. Ingenuity Pathway Analysis obtained from SKOV3 following MSC contact.

Chemotherapeutic treatment of OCC induced 65% of cell death, while co-culture with MSC rescued OCC with 1.6 fold decrease in cell death [14]. Similarly as previously IPA network analyses suggest that OVCAR3 chemoresistance appears to be mediated through up-regulation of genes such as GADD45A, DDIT3, NR3C1, ATF2, and RASGRP1 (Figure 4A). SKOV3 cell line down-regulated the following genes implicated in apoptosis BTG2, SCD, FASN, DDIT4 and up-regulated survival genes such as SPARC, PDGFRA, S1PR3, KITLG, IGFBP (Figure 4B).

### SKOV3 co-cultured with MSC resemble some human tumors

**Figure 5:**
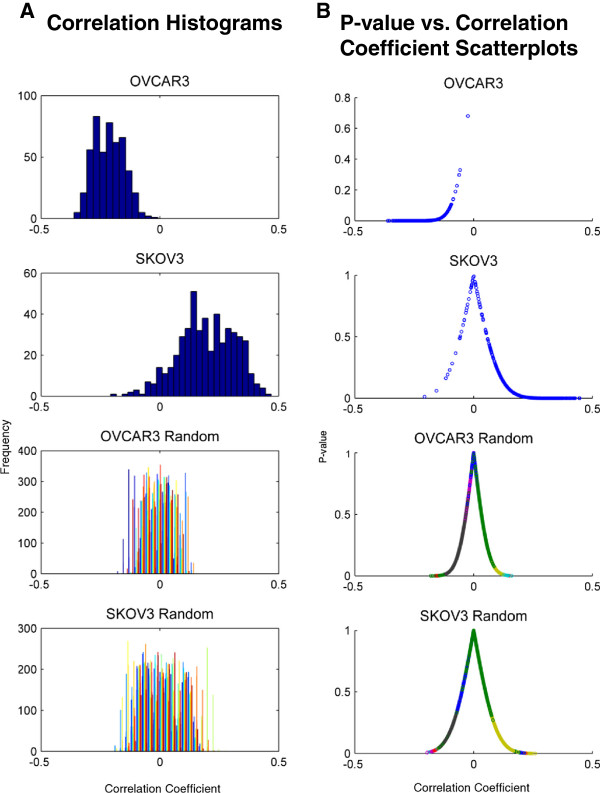
**Correlation of OVCAR3 and SKOV3 gene expression to patient tumors. A)** Histograms of the correlation coefficients, calculated between every patient gene expression profile and cell line gene expression profiles, show that OVCAR3 expression following MSC coculture is negatively correlated with patient tumor expression while SKOV3 expression following MSC coculture is positively correlated. The OVCAR3 and SKOV3 random correlation distribution as shown by the random histograms indicates that the high positive and negative correlations are not due to chance. **B)** Scatterplots showing the p-value variation with the correlation coefficient indicates that the high and low correlations have significantly low p-values. P-value scatterplots of the random correlations also show higher p-values than the sample correlations indicating significant sample correlations.

To determine whether the highly significant expression changes seen after cell lines are co-cultured with MSCs could have some clinical relevance, we calculated the similarity (Pearson’s correlation coefficient) between the expression changes for each cell line and publically available human ovarian cancer expression data from 493 patients. There were 293 OVCAR3 genes and 143 SKOV3 genes for which correlations were calculated with the patient data. We find (Figure 5) that SKOV3 is positively correlated with the tumor expression data while OVCAR3 is negatively correlated. These correlations for both cell lines are highly significant both in terms of the associated p-value for the correlation and in terms of not resembling correlations expected by random. This observation suggests that SKOV3 cells after treatment with MSCs results in gene expression changes that resemble human tumors. In contrast, OVCAR3 gene expression changes do not resemble the TCGA human tumors.

## Discussion

In this follow-up study we show that despite similar phenotypic modulation different cell lines of a similar cancer type display diverse transcriptomic modifications. The acquisition of an increased metastatic phenotype during the cross talk between cancer and stromal cells is concordant with previous report in ovarian as well as other tumor types [5,6,9,10,15]. Interestingly our approach using two different cell lines associated to a network analysis illustrate that the shift of phenotype will be dependent on the transcriptomic background. This is quite relevant in the context of targeted therapies. The network analysis presented above allows a more detailed interpretation of the relationship between transcriptomic pro-metastatic changes in OCC and differences in cell behavior upon co-culture for the different functionality tested.

### Migration, invasion and adherence

Increased adherence and invasion constitute a key step toward peritoneal metastasis [2]. Both cell lines regulated genes related to these processes; interestingly some had opposite regulation. FN1 encoding fibronectin was up-regulated (2-fold) in SKOV3, and down-regulated (5.63-fold) in OVCAR3. As demonstrated by different studies Fibronectin does not trigger similar response in the two cell lines studied. In contrast to SKOV-3, OVCAR-3 cells did not migrate or invade matrigel either with or without FN [16,17]. OVCAR3 cell line up-regulation of Twist and Zeb indicates an epithelial to mesenchymal transition state. The expression of E-cadherin in ovarian cancers plays a role in the adhesion to the peritoneal surfaces. The ovarian surface epithelium (OSE) does not express E-cadherin, but its expression appears in inclusion cysts of the ovary and is reinforced during tumoral progression [18]. One might argue against simultaneous up-regulation of EMT markers (TWIST, ZEB) and up-regulation of E-cadherin and downregulation of FN1. However recently a subpopulation of cells in transitory Epithelial/Mesenchymal stage presenting increased tumorigenicity has been described [19]. The described cell plasticity appeared to be dependent on external factors such as co-culture with mesenchymal cells. Finally the mixed state might represent a transient state induced by the co-culture setting. SKOV3 cells displayed up-regulation of MMP3 concordant with previous reports [20]. Several pro-metastatic cytokines already described were up-regulated such as CXCR4, and CCL2 [21,22]. Hence, our strategy to build focused networks for specific functionalities allows understanding their inter-relationship and thereby might help designing better disrupting strategies.

### Proliferation

OVCAR3 proliferation relied on NR3C1 node that has already been described associated with liver metastasis of gastric cancer [23]. PRKACB stabilizes elF4F expressed at high level in different carcinomas. RhoB functions as tumor suppressor genes and its down-regualtion promotes cancer cell proliferation [24]. CEBPB is involved in transcriptional up-regulation of SphK1 by LPA in gastric cancers [25]. Another interesting function of CEBPB is its role in the occurrence of mesenchymal phenotype in brain tumors where it is a synergistic initiator of mesenchymal transformation with STAT3 [26]. VEGF has a role in increased proliferation and resistance to anoikis of ovarian carcinomas beside its role in angiogenesis [27]. Finally SPARC modulates cell-cell and cell-matrix interactions and demonstrates de-adhesive and growth inhibitory properties in non-transformed cells. Recently Said et al. showed that SPARC significantly inhibited OVCAR3 basal and LPA-induced interleukin IL6 production and attenuated IL-6-induced mitogenic, chemotactic, and proinvasive properties through ERK1/2 inhibition [28,29]. Once again interestingly SPARC was up-regulated in SKOV3. Increased SPARC is associated to poor prognosis in pancreatic adenocarcinoma and to lymph node metastasis in gastric cancers [30]. In melanoma cells SPARC promotes proliferation and survival through akt-dependant regulation of p53 [31]. In a three dimensional context the interaction of fibronectin and alphaV integrin has been associated to increased proliferation in ductal pancreatic carcinoma [32]. In a model of lung carcinoma, inhibition of the interaction between fibronectin and alphaV integrin resulted in reduced proliferation [33].

### Chemoresistance

While many genes for both cell lines are similar to the ones responsible for increased proliferation the central nodes are not always the same suggesting the ability of cancer cells to use complex transcriptomic machinery to respond to cytotoxic stress. OVCAR3 displayed increased GADD. The deficiency of gadd45 increases cell sensitivity to UV irradiation or cisplatin [34,35]. Fibronectin was found to be central for SKOV3 chemoresistance. Fibronectin ligand integrin alphaVbeta1 plays a role in multicellular aggregation and resistance to paclitaxel [36]. Moreover activation of alphaVbeta1 activates GSK3β pro-survival signaling [37]. Finally fibronectin mediated adhesion promotes Akt phosphorylation in highly metastatic cancer cells A2780 and MDA-MB231, and further induced chemoresistance against docetaxel [38,39].

Other genes have been described in the literature in ovarian cancer biology. However they shall not be uncovered in our approach if they do not play a role in the co-culture context [7,40,41]. It is hard to interpret all the genes described in the networks in a cancer-cell autonomous context. Some genes might not have the expected regulation but might play a role in the cross-talk between OCC and MSC.

As recently illustrated in oncogenomic approaches the role of a single genes might not be as important as pathways up or down-regulated. Our approach uncovered some central nodes (such as fibronectin) and their role will be analyzed in separate targeted functional studies.

The main question raised in this research was if broad transcriptmic changes underlie phenotypic modulation in cancer cells co-cultured with mesenchymal stem cells. We have thus chosen the following experimental approach. We have co-cultured for 24 hours two different cancer cell lines and mesenchymal cells and performed cell sorting and transcriptomic analysis at a time-point where we observed functional effect of the co-culture. We then used targeted network analysis of the most significant shifted genes and determined few networks described above.

While we acknowledge that validation is an important part of such studies but there are many studies where the role of single genes or few genes were validated without any translational relevance. Here we do not want to demonstrate the role of any particular genes but we want to emphasize that the interaction in vitro between stromal and cancer cells induces broad transcriptomic profile that should be carefully considered when experimental plans are designed. Interestingly showing differential expression of genes between two routinely used ovarian cancer cells indicate that one must be quite careful before investigating the particular role of a single gene.

### Clinical implications

Our observation that one cell line can shift expression to more resemble human tumors is interesting but must be examined more closely. It is interesting because it suggests that culturing MSCs with tumor cells can create a better model system to study carcinogenesis since the co-cultured tumors cells bear more resemblance to human tumors. It is also interesting that this effect is cell line dependent as OVCAR3 shifts farther away from human tumors after co-culture with MSC’s. However, while this result is interesting, it should be seen in the context that it is difficult to compare expression data obtained at different times and with different platforms. Nevertheless, this observation merits further investigation.

Other ovarian cancer cell lines need to be tested co-cultured with different cells to obtain a broader picture of the role of the genetic background in the interaction with the microenvironment. Understanding the differences between ovarian serous adenocarcinoma types and their interaction with the microenvironment would open the path to new personalized therapeutic strategies.

## Conclusion

Here we demonstrate that transcriptomic changes induced by MSC co-culture were cell type specific for the two cell lines investigated here. Many studies suggest the role of the tumor stroma in disease progression as well as residual disease. Understanding the interactome between the cancer and stromal cells will lead to new stroma specific therapeutic disrupting pro-tumoral cross talk. We have to consider tumor heterogeneity and therefore differential plasticity both in location and time to tailor treatments.

## Competing interests

The authors declare that they have no competing interests.

## Authors’ contributions

Conception and design: RL, CT and ART. Acquisition of data: RL, CT, AS. Analysis and interpretation of the data: RL, CT, NMH, AS, JM, JAM, KS, DQ and ART. Manuscript preparation: RL, CT, NMH and ART wrote the manuscript. Manuscript reviewing: RL, CT, DQ, NMH and ART. All authors read and approved the final manuscript.

## Supplementary Material

Additional file 1 Gene expression extracted from the TCGA data sets. Click here for file

Additional file 2 Transcriptomic modification of SKOV3 and OVCAR3 after co-culture with MSCs. Click here for file

Additional file 3: Figure S1 Genes network obtained from OVCAR3-eGFP following MSC contact using all genes included in the Ingenuity Pathway Anlaysis category “Cancer”. Click here for file

Additional file 4: Figure S2 Genes network obtained from SKOV3-eGFP following MSC contact using all genes included in the Ingenuity Pathway Anlaysis category “Cancer”. Click here for file
