# Supplementary figures and images for: Mesenchymal cell interaction with ovarian cancer cells induces a background dependent pro-metastatic transcriptomic profile
Source: J Transl Med. 2014 Mar 5;12:59. doi: 10.1186/1479-5876-12-59 (PMC4132214; doi:10.1186/1479-5876-12-59)

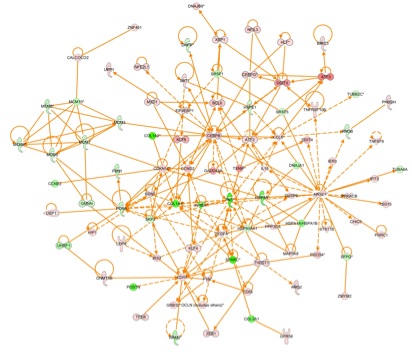

Supplement: Additional file 3: Figure S1 — Genes network obtained from OVCAR3-eGFP following MSC contact using all genes included in the Ingenuity Pathway Anlaysis category “Cancer”. [file 1479-5876-12-59-S3.jpeg]

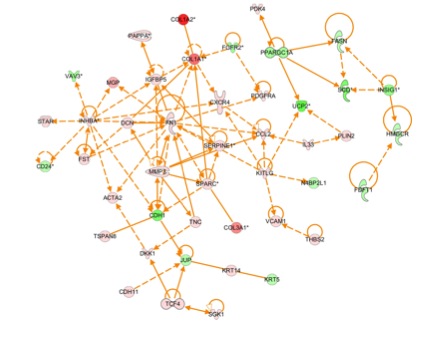

Supplement: Additional file 4: Figure S2 — Genes network obtained from SKOV3-eGFP following MSC contact using all genes included in the Ingenuity Pathway Anlaysis category “Cancer”. [file 1479-5876-12-59-S4.jpeg]
